# Monitoring the Molecular Conformation of Individual Amphotericin B Molecules in an Aggregated State by Raman Optical Activity

**DOI:** 10.1021/acs.analchem.5c01198

**Published:** 2025-05-28

**Authors:** Katarzyna Pajor, Grzegorz Zając, Marco Fusè, Marzena Mach-Liszka, Marta Arczewska, Mariusz Gagoś, Yoshimitsu Onaka, Tomotsumi Fujisawa, Masashi Unno, Malgorzata Baranska, Ewa Machalska

**Affiliations:** a Faculty of Chemistry, 37799 Jagiellonian University, Gronostajowa 2, 30-387 Krakow, Poland; b Doctoral School of Exact and Natural Sciences, Prof. S. Lojasiewicza 11, 30-348 Krakow, Poland; c Jagiellonian Centre for Experimental Therapeutics (JCET), Jagiellonian University, Bobrzynskiego 14, 30-348 Krakow, Poland; d Department of Molecular and Translational Medicine, Università di Brescia, 11 Viale Europa, 25123 Brescia, Italy; e Department of Biophysics, University of Life Sciences, Akademicka 13, 20-033 Lublin, Poland; f Department of Cell Biology, Maria Curie-Sklodowska University, Akademicka 19, 20-033 Lublin, Poland; g Department of Chemistry and Applied Chemistry, Faculty of Science and Engineering, 13030 Saga University, Honjo-machi 1, Saga 840-8502, Japan; h Laboratory for Spectroscopy, Molecular Modeling and Structure Determination, Institute of Nuclear Chemistry and Technology, Dorodna 16, 03-195 Warsaw, Poland

## Abstract

The polyene antifungal amphotericin B (AmB) can form giant helical aggregates. We show that microscopic and mesoscopic structural features of its aggregates can be revealed by Raman optical activity (ROA) and electronic circular dichroism (ECD), respectively. The ROA method, which senses molecular structure more locally, elucidates the conformation of the polyene chain of individual AmB molecules in an aggregated state. In turn, the ECD signal related to exciton coupling effects provides details about the arrangement of AmB molecules in the supramolecular structure. Thus, the use of both complementary methods will be crucial in future structural studies of chiral supramolecular systems.

## Graphical abstract

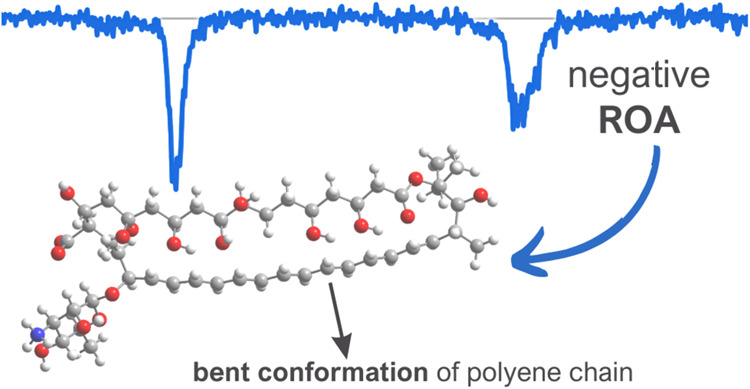

## Introduction

The detailed structural characterization of chiral supramolecular architectures is important in numerous research areas, ranging from biomolecular chemistry^1^ and asymmetric catalysis^2^ to functional materials.^3^ Notably, chiroptical spectroscopies are powerful tools for determining the structures of supramolecular assemblies.^4–6^ Among these methods, the exciton coupling effect used in electronic circular dichroism (ECD) spectroscopy is the most popular for analyzing aggregate states.^7^ However, because the effect of exciton coupling is usually too strong, it is practically impossible to obtain the local molecular structure within an aggregate from the ECD signal. In this study, we solved this issue by using Raman optical activity (ROA). When combined with quantum chemical calculations, ROA has proven to be a reliable and convenient approach to provide detailed information regarding the absolute molecular configuration,^8^ conformations,^9^ and chirality of biologically active molecules in solution.^10,11^ Here, we present the utility of ROA spectroscopy in determining the molecular conformation of an individual molecule in an aggregate. For this purpose, we selected amphotericin B (AmB), a large and flexible molecule of medical significance. AmB is an effective yet toxic polyene antibiotic used to treat invasive and rare fungal diseases (Figure 1).^12,13^

**Figure 1:**
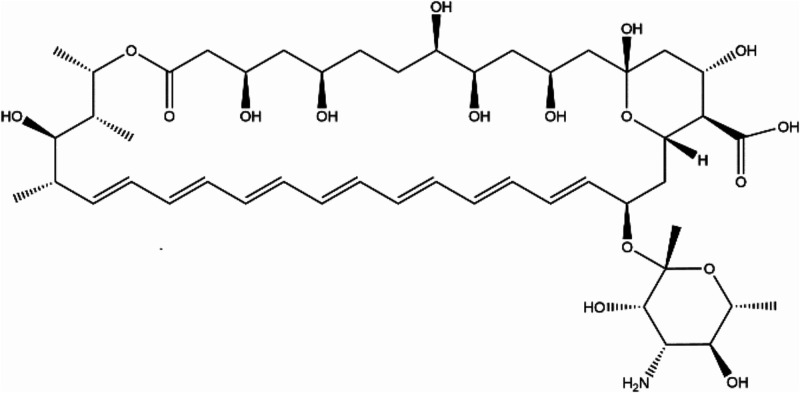
Molecular structure of amphotericin B (AmB).

The toxicity of AmB is primarily linked to its aggregation state.^14,15^ While water-soluble dimers of AmB are toxic due to high cholesterol affinity, the safety levels of superaggregates^16,17^ are similar to those of ergosterol-specific monomers.^18–21^ In particular, high aggregation levels of AmB were observed in model systems for the compound dissolved in water solution at pH values below 2,^22^ at 12,^23^ or under high temperatures.^16,24^ This kind of biomolecular aggregation usually results in changes in ultraviolet–visible (UV–vis) absorption and ECD spectra, which are sensitive to the type of aggregate.^25,26^ Namely, polyene molecules can form ordered supramolecular assemblies, i.e., tightly packed H-aggregates characterized by blue-shifted UV–vis signals and weakly coupled J-aggregates revealing red shifts of electronic absorption.^27^

Here, we should note that several ECD and ROA measurements have been reported under resonance^25,26,28–30^ and preresonance^31^ conditions for similar polyene systems, specifically, carotenoid aggregates and monomers. After carotenoid aggregation, a significant induction of both ECD and pre- or resonance ROA signals was observed, and this effect is known as the aggregation-induced preresonance (pRROA)^31^ or resonance ROA (AIRROA).^25,26,28–30^ However, since carotenoid monomers do not exhibit a pronounced ECD signal in the visible region, measuring RROA using a green laser line (532 nm) was practically impossible.

The above observation clearly differs from the present case on AmB, as we will show below. In this contribution, we demonstrate that combining ECD and ROA spectroscopies can characterize the supramolecular arrangement and individual molecules in the aggregated state. Markedly, a strong ECD signal based on an exciton coupling mechanism (the Cotton effect) provides direct insight into the helical organization of AmB molecules. In turn, ROA spectroscopy, which senses a molecular structure more locally, can be used to examine the conformation of the polyene chain in the individual AmB molecule within the complex arrangement.

## Experimental Section

### Materials and Reagents

All reagents, i.e., AmB from Streptomyces sp., dimethyl sulfoxide (DMSO), methanol (MeOH), 2-propanol, and sodium hydroxide (NaOH) were purchased from Sigma-Aldrich. To remove microcrystals of the remaining AmB in the sample, the compound was dissolved in 40% 2-propanol and then centrifuged for 15 min at 15,000*g*. AmB was further purified using HPLC on a YMC C-30 coated phase reversed column (length 250 mm, internal diameter 4.6 mm) with 40% 2-propanol in H_2_O as a mobile phase. The final concentration of AmB was calculated from the absorption spectra using the molar extinction coefficient 1.3 × 10^5^ M^–1^ cm^–1^ given for the UV–vis band at ∼410 nm. After drying, the AmB was stored under argon in a fridge (approximately 4 °C).

### Measurements

UV–vis and ECD spectra of AmB solutions were measured on the Jasco J–815 spectropolarimeter in 50, 10, 1, 0.1, or 0.01 mm optical cells with an accumulation of 1–40 scans, a scanning speed of 100 nm min^–1^, a step size of 0.1 nm, a bandwidth of 1 nm, and a response time of 1 s. The spectra were background-corrected using the respective solvent measured under the same conditions (Figures 2, S1–S6).

**Figure 2:**
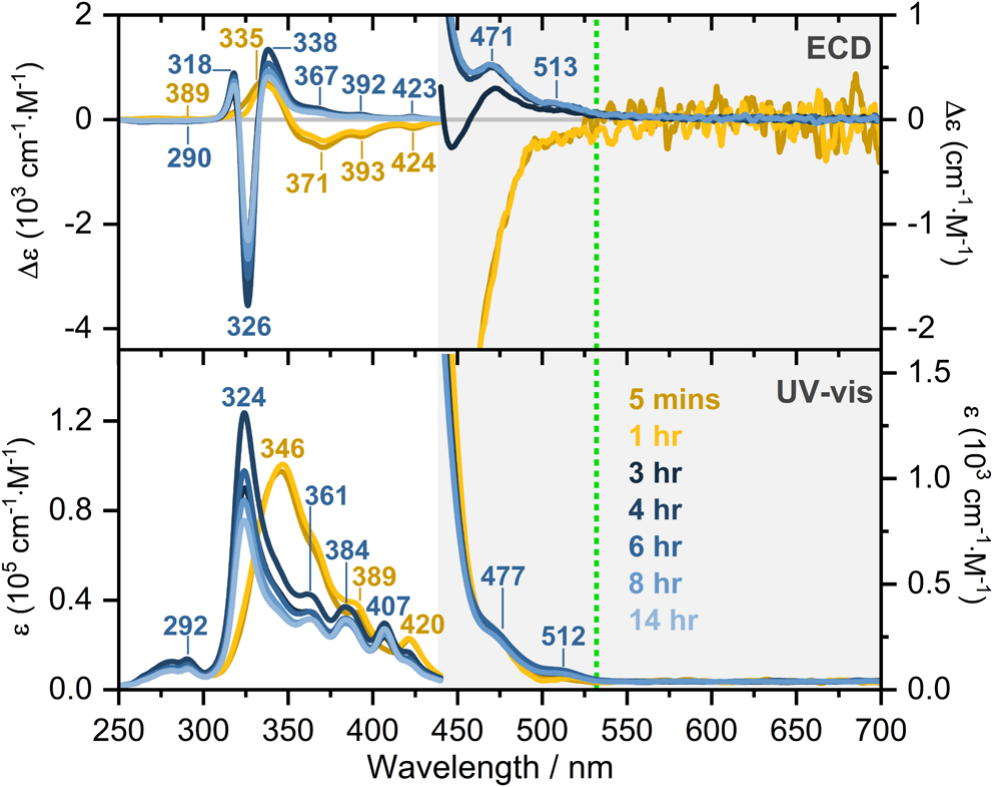
ECD and UV–vis signals obtained for AmB species in alkaline water (pH 12.7) at a concentration of 8 × 10^–3^ M. The electronic spectra were measured after 5 min, 1, 3, 4, 6, 8, and 14 h of the sample preparation. The green line indicates the excitation line used to register RR and RROA spectra (532 nm).

Raman (RR) and Raman optical activity (RROA) spectra of AmB solutions in resonance conditions were recorded using ChiralRAMAN-2X spectrometer (BioTools Inc.) equipped with the excitation wavelength of 532 nm, within the 2500–250 cm^–1^ spectral range, with a resolution of 7 cm^–1^ and an integration time of 1 s. On the other hand, preresonance Raman (pre-RR) and ROA (pre-RROA) spectra were measured on a home-built ROA spectrometer^32,33^ at 12.5 cm^–1^ spectral resolution in the range of 1900–200 cm^–1^ using the 785 nm laser source. Other experimental terms, i.e., concentration, solvent, laser power, and data collection time, were listed in Table S1. To avoid laser-induced decomposition of AmB samples, the RR and RROA spectra were collected employing low laser powers in a temperature controller operating at 20 °C. Each vibrational spectrum was confirmed by at least two experiments obtained from independently prepared samples. Note that the spectra of AmB in an aqueous solution were measured after 6 h of sample preparation.

### Quantum Chemical Calculation

Density functional theory (DFT) calculations were performed using Gaussian 16.^34^ The crystal structure (PDB: 7SHI) of the protein-bound AmB was used as the initial starting geometry.^35^ A geometry optimization was performed at B3LYP/6–31G* level of theory using the CPCM polarizable conductor model with DMSO solvent parameters set. The same level of theory was used to compute Raman intensity with 785 nm excitation.

### MD and QM/MM Calculations

The molecular dynamics (MD) and quantum mechanics/molecular mechanics (QM/MM) calculations were carried out as described previously.^36^ MD simulation was performed with the Amber16 program^37^ using an explicit representation of solvent (DMSO) molecules^38^ and the all-atom force field for AmB, which was generated by an Antechamber program.^37^ Geometry was taken from the crystal structure of AmB in a protein (PDB: 7SHI), and the AmB molecule was surrounded by a periodic rectangular box of 325 DMSO molecules. The whole system was subjected to minimization and heated to 300 K by a 20 ps MD run as described.^36^ The system obtained after heating was simulated for 500 ns at 1 atm.

The subsequent QM/MM calculations were performed using the Gaussian16 suite of programs.^34^ A two-layer ONIOM method was used to perform the QM/MM calculations. The initial geometries were obtained from snapshots of the MD simulation, and 50 DMSO molecules near the solute molecule were included. The QM region consists of AmB and was treated by DFT. The remainder of the system was treated as the MM region, which was described with the Amber force field. The QM part of the system was computed at the B3LYP/6–31G* level of theory. An electronic embedding scheme that considers the partial charges of the MM region into the QM Hamiltonian was used. The calculated spectra were obtained assuming a Gaussian band shape with a half-width of 10 cm^–1^. The calculated vibrational frequencies were uniformly scaled using a factor of 0.9623.

### ECD-Raman Correction

ECD-Raman correction was done according to the already described procedure^39,40^ (SI).

## Results and Discussion

Figure 2 illustrates the UV–vis and ECD spectra of AmB species recorded at a pH of 12.7 and a concentration of 8 × 10^–3^ M. In agreement with previous results,^23^ AmB monomers undergo spontaneous self-association over time in an alkaline solution. Indeed, the time dependence of ECD and UV–vis signatures was detected, and the signals stabilized 6 h after sample preparation. A blue shift of the S_0_(^1^1 Ag)→S_2_(^1^1 Bu) transition^20^ from 346 to 324 nm occurs, and the ECD spectrum gains intensity, showing a distinctive +/–/+ set of features at 338, 326, and 318 nm. These spectral changes were attributed to the formation of the giant helical H-aggregates with a left-handed helical organization of AmB molecules.^23,41,42^ The tight packing of H-type aggregates, along with high concentration, protects the AmB polyene moiety against oxidation.^23,43^ The spectral trends of AmB in aqueous solution at concentrations of 8 and 4 × 10^–3^ M were identical (Figure S3). In contrast, as shown recently,^20^ only slight changes in UV–vis and ECD spectra occur over time for AmB in DMSO and DMSO/MeOH mixtures (Figures S4, S6). This latter observation indicates that AmB exists as a monomer in the organic solvents and their mixtures.

To examine the impact of aggregate formation on the structure of individual AmB molecules, we measured the Raman and ROA spectra. The experimental Raman spectra, collected using 785 and 532 nm laser lines, are shown in the upper part of Figure 3. Under both preresonance and resonance conditions, the Raman spectra are noticeably simplified, with enhanced bands corresponding to the vibrations of the polyene chain. Consequently, the Raman spectra of AmB, measured in aqueous solutions and organic solvents, are dominated by two intense, well-resolved signals in the 800–1800 cm^–1^ range. These bands are assigned to the C=C stretching mode (∼1560 cm^–1^) and the C–C stretching mode coupled with C–C–H bending vibrations (∼1155 cm^–1^).^44^

**Figure 3:**
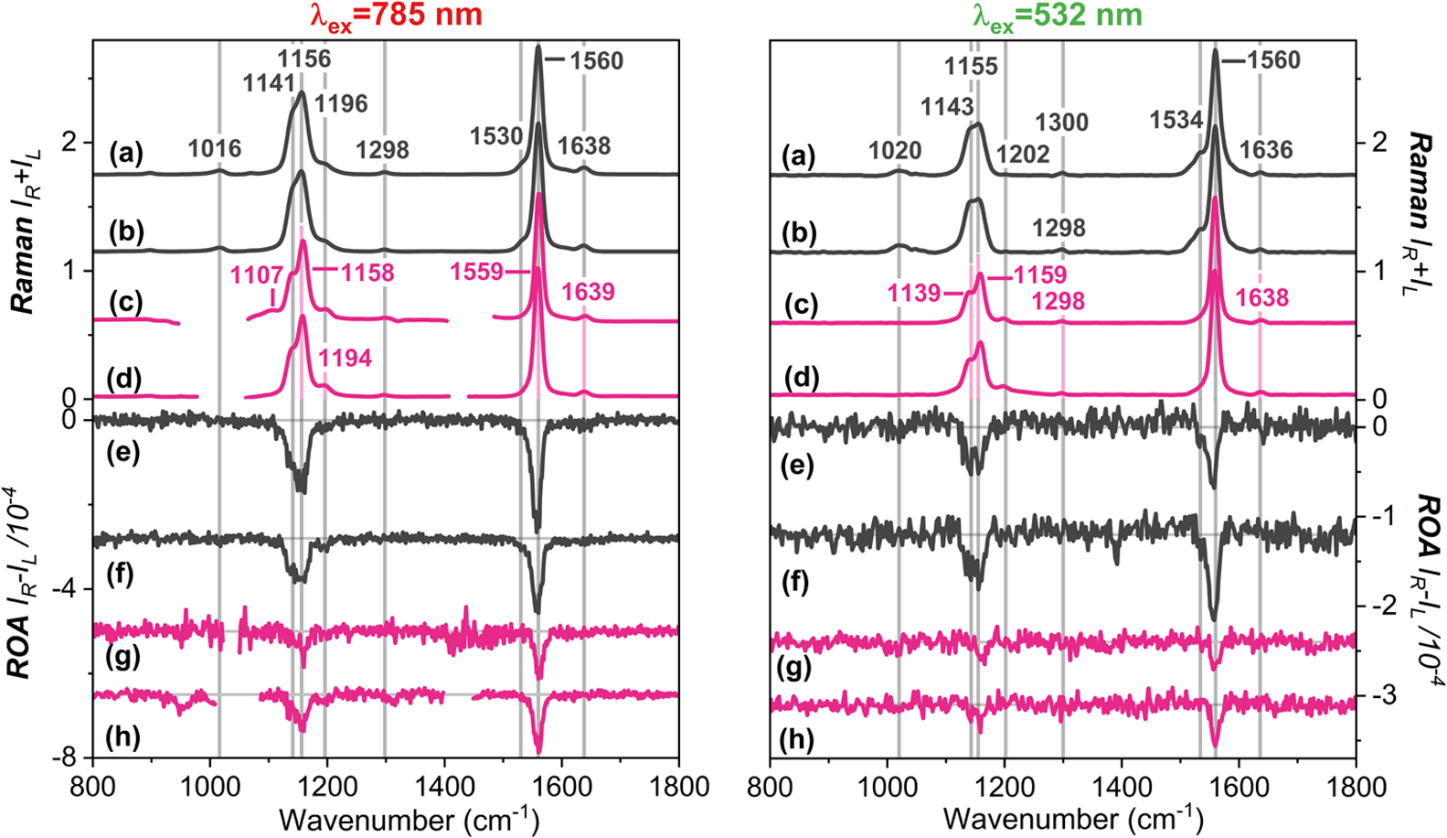
Comparison of Raman and ROA spectra of AmB in alkaline water (pH 12.7) at concentrations of 4 × 10^–3^ M (**a, e**) and 8 × 10^–3^ M (**b, f**); in a 1:1 (v/v) mixture of DMSO/MeOH (**c, g**); and DMSO (**d, h**) at 8 × 10^–4^ M. The spectra were collected using 785 and 532 nm excitation wavelengths and normalized to the most intense band at ∼1560 cm^–1^.

Figure 3 shows that RR and pre-RR spectra of monomeric AmB in DMSO/MeOH (trace **c**) and DMSO (trace **d**) are almost identical to each other in the entire spectral range, similar to what is observed in their electronic spectra (Figure S4). The figure also displays that the spectral signatures of AmB in organic solvents (traces **c** and **d**) and alkaline solutions (traces **a** and **b**) remain similar to each other. These results for the Raman spectra differ from those for the electronic spectra, which exhibit significant changes upon aggregate formation. However, a careful inspection reveals some differences. These include a band at ∼1155 cm^–1^ with a well-defined shoulder at lower energies (∼1140 cm^–1^), attributed to C–C–H bending and C=C–C distortion motions.^44^ Notably, spectral differences also depend on the excitation laser line. This effect is associated with increased relative intensities of vibrational bands (mostly the shoulder at ∼1140 cm^–1^ in AmB aqueous solution) and a less pronounced fluorescence background (Figures S7–S8) in pre-RR spectra compared to those recorded under resonance conditions.

The lower parts of Figure 3 present the experimental RROA and pre-RROA spectra. Despite using two different laser excitations, vibrational spectra were successfully recorded at the same AmB concentrations, which is crucial for studying supramolecular systems. Both excitation wavelengths produce monosignate signatures with two prominent negative bands (∼1560 and ∼1155 cm^–1^), associated primarily with the stretching motions of the polyene chain. As predicted by RROA theory under the single electronic state (SES) limit, the relative intensities of the RROA signals are similar to those of the RR counterpart, and the sign of the RROA bands is opposite to that of the ECD band in resonance.^33,45^ Specifically, the negative RROA spectral pattern arises from the S_0_→S_2_ electronic transition, which corresponds to a positive ECD signal, with a maximum at ∼340 nm and distinct vibrational substructures at 513, 471, 423, 392, and 367 nm (Figure 2).

The exciton splitting manifested in the ECD spectrum hides the intrinsic ECD signal of the individual AmB molecule in the aggregate. However, ROA senses subtle changes in molecular structure locally and, more precisely, monitors conformations. To obtain structural information from the observed Raman and ROA spectra, we have performed QM/MM calculations combined with MD simulations. A 500 ns MD run at 300 K was performed using the crystal structure of AmB in a protein^35^ as an initial starting geometry and including explicit DMSO solvent molecules. The 51 snapshots of the 500 ns simulations were taken every 10 ns for the subsequent QM/MM calculations. One structure and the conformational distribution of the QM/MM optimized structures are shown in Figure S9. To represent the flexibility of the polyene chain, we utilized two geometrical descriptors: the C20–C33 distance as a measure of polyene chain bending (panel A), and the dihedral angles between the planes defined by C20–C21–C22 and C31–C32–C33 as a measure of its twisting (panel B). Although the figure clearly shows conformational fluctuations, the averaged structure of AmB adopts a slightly bent and twisted conformation.

Next, we carried out the QM/MM calculations to simulate the Raman and ROA spectra of AmB in DMSO (Figure 4). The simulated spectra closely replicated the experimental spectral features, showing a small negative circular intensity difference (CID, the ratio of ROA to Raman; Table S3) consistent with observations in DMSO or DMSO/MeOH mixture.

**Figure 4:**
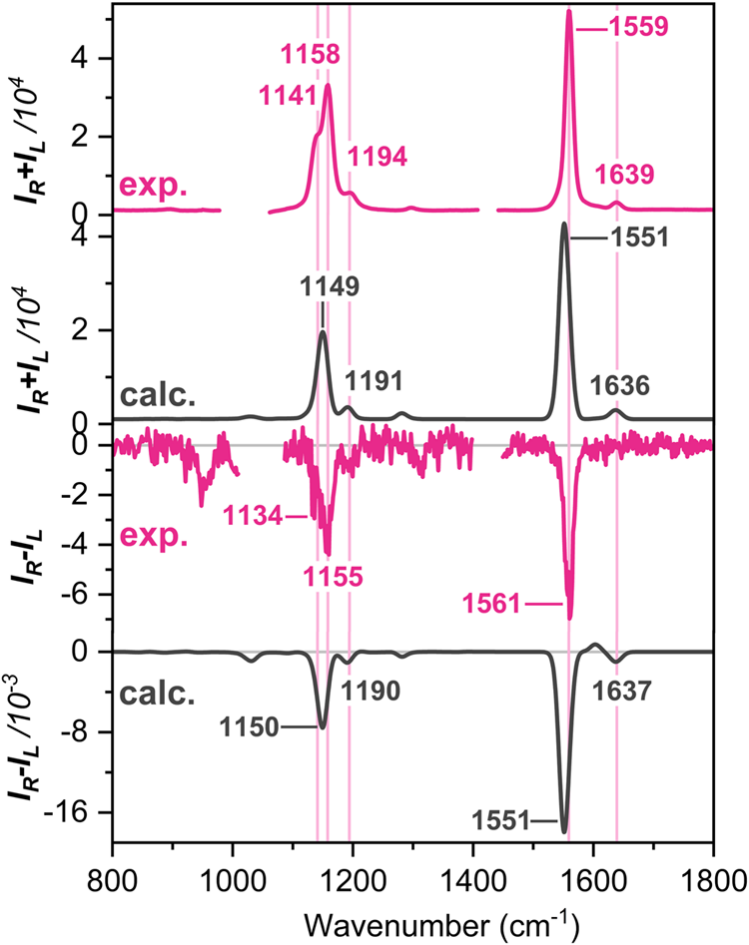
Comparison of the experimental (pink line) and simulated (black line) Raman and ROA spectra of AmB in DMSO. All spectra were obtained using an excitation wavelength of 785 nm. The calculated spectra were averaged over 51 MD clusters.

As depicted in Figure 3, AmB aggregates in alkaline water exhibit larger ROA intensities compared to molecular systems in the organic environment. This result suggests a more distorted chiral conformation of the polyene moiety in the supramolecular structure. A previous study^23^ proposed that the polyene moiety of AmB may adopt a twisted conformation in the aggregate state. In fact, our previous report demonstrated that a left-handed helical twist of the polyene moiety generates the negative ROA signal.^46^ Moreover, Figure 5 presents the time dependence of the pre-RR and pre-RROA spectra of AmB species in alkaline water at a concentration of 4 × 10^–3^ M. Comparison of traces **a** (0–15 h), **b** (15–30 h), and **c** (30–45 h) reveals that the intensities of the pre-RR spectra gradually increase. At the same time, in both pre-RR and pre-RROA spectra, the band shapes in the 1100–1200 cm^–1^ range change over time. This spectral evolution clearly indicates that aggregate formation influences the structure of individual AmB molecules. Further investigation of the structural changes associated with aggregate formation will be the focus of our future studies.

**Figure 5:**
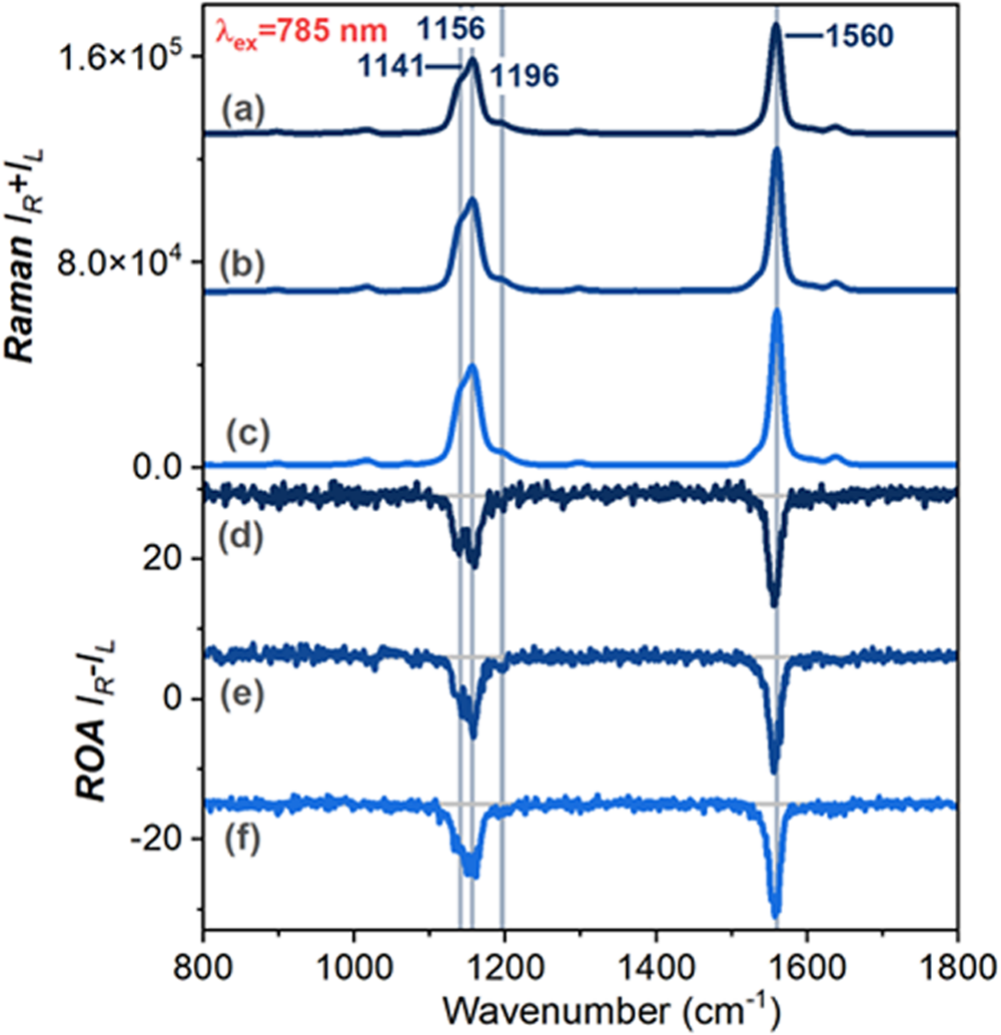
Time-dependent Raman and ROA spectra of AmB measured at pH 12.7 and a concentration of 4 × 10^–3^ M over 0–15 (**a, d**), 15–30 (**b, e**), and 30–45 h (**c, f**). The spectra were collected using a 785 nm excitation wavelength and normalized to the intense band at ∼1560 cm^–1^.

The CID (Table S3) values differ at different excitation lines (532 and 785 nm). As mentioned above, the negative monosignate patterns in the ROA spectra of AmB are consistent with RROA theory under an SES limit. However, the CID values of the two most intense signals show higher absolute values in pre-RR conditions than in the RR regime. For instance, for the C=C stretching mode (∼1560 cm^–1^) of AmB at a concentration of 8 × 10^–3^ M in an alkaline medium, the CID values are −9.8 × 10^–5^ and −1.7 × 10^–4^ for the RR and pre-RR conditions, respectively. This observation appears contradictory, as CID values are expected to decrease with increasing excitation wavelength.^47^ In fact, the DFT calculations predicted smaller CID values under pre-RR conditions, although the different excitation wavelengths little affect the Raman and ROA spectral features (Figure S11). Furthermore, the observed trend for AmB contrasts with that noted for carotenoid species. Generally, their CID values were rather comparable to the ECD/UV–vis ratio, thus being twice the value predicted by the SES theory.^25^

The ECD-Raman effect^39,40^ may also influence the signs and intensities of RROA signals, as the chiroptical signal may be a sum of the natural RROA and ECD-Raman components. However, in our experiments, ECD-Raman interference was subtracted from the RROA spectra (Figure S7). For AmB species, the calculated ECD-Raman effect is negligible, primarily due to very low absorption in the 532–584 nm range, which corresponds to 0–1700 cm^–1^ of the Raman shift when using the 532 nm excitation line.

## Conclusions

In summary, this study demonstrates, for the first time, the potential of ROA spectroscopy in characterizing the molecular conformation of individual AmB molecules in an aggregated state. The strong exciton Cotton effects typically mask the intrinsic CD signal of the AmB molecule. However, ROA spectral features reveal conformational changes in the chromophoric polyene chain. Such structural information, rarely accessible via UV–vis and ECD spectroscopy, was successfully obtained through ROA. These insights into AmB conformation contribute to a deeper understanding of its therapeutic antifungal effects.

## Supplementary Material



## Notes

Additional experimental details along with the electronic and vibrational spectra (PDF)
