# Supplementary material for: Monitoring the Molecular Conformation of Individual Amphotericin B Molecules in an Aggregated State by Raman Optical Activity
Source: Anal Chem. 2025 May 28;97(22):11754–9. doi: 10.1021/acs.analchem.5c01198 (PMC12163884; doi:10.1021/acs.analchem.5c01198)
Supplement: Supplementary file 1 [file ac5c01198_si_001.pdf]

SUPPLEMENTARY INFORMATION TO

Monitoring the molecular conformation of individual amphotericin B molecules in an aggregated state by Raman optical activity

Katarzyna Pajor [a,b], *Grzegorz Zając* [c], *Marco Fusè* [d], *Marzena Mach-Liszka* [a], *Marta Arczewska* [e], *Mariusz Gagoś* [f], *Yoshimitsu Onaka* [g], *Tomotsumi Fujisawa* [g], *Masashi Unno** [g], *Malgorzata Baranska* [a], *Ewa Machalska** [c,d,h]

[a] *Faculty of Chemistry, Jagiellonian University, Gronostajowa 2, 30-387 Krakow, Poland*

[b] *Doctoral School of Exact and Natural Sciences, Prof. S. Lojasiewicza 11, 30-348 Krakow, Poland*

[c] *Jagiellonian Centre for Experimental Therapeutics (JCET), Jagiellonian University, Bobrzynskiego 14, 30-348 Krakow, Poland*

[d] *Department of Molecular and Translational Medicine, Università di Brescia, 11 Viale Europa, 25123 Brescia, Italy, e-mail: ewa.machalska@unibs.it*

[e] *Department of Biophysics, University of Life Sciences, Akademicka 13, 20-033 Lublin, Poland*

[f] *Department of Cell Biology, Maria Curie-Sklodowska University, Akademicka 19, 20-033 Lublin, Poland*

[g] *Department of Chemistry and Applied Chemistry, Faculty of Science and Engineering, Saga University, Honjo-machi 1, Saga 840-8502, Japan, e-mail: unno@cc.saga-u.ac.jp*

[h] *Laboratory for Spectroscopy, Molecular Modeling and Structure Determination, Institute of Nuclear Chemistry and Technology, Dorodna 16, 03-195 Warsaw, Poland*

TABLE OF CONTENTS

Table S1. Set of Raman and ROA measurement parameters of AmB solutions S3
ECD-Raman correction S3
Figure S1. Comparison of ECD and UV-vis spectra of AmB in water at pH=12.7 in various concentrations S4
Figure S2. Comparison of ECD and UV-vis spectra of AmB ($4x10^{-3}$ M) in water at pH=12.7 measured 5 minutes, 1, 3, 4, 6, 8, and 14 hours after the sample preparation S4
Figure S3. Comparison of ECD spectra of AmB ($4x10^{-3}$ and $8x10^{-3}$ M) in water at pH=12.7 measured 6 and 24 hours after sample preparations S5
Figure S4. Comparison of ECD and UV-vis spectra of AmB ($8x10^{-4}$ M) in DMSO and a mixture of DMSO/MeOH S5
Figure S5. Comparison of ECD and UV-vis spectra of AmB in DMSO in various concentrations S6
Figure S6. Comparison of ECD spectra of AmB in DMSO measured 5 minutes, 6, and 9 hours after sample preparation S6
Figure S7. Comparison of solvent-corrected RR and RROA spectra (λ_{ex}=532 nm) and subtracted ECD-Raman effect of AmB in organic and inorganic solvents S7
Figure S8. Comparison of solvent-corrected pre-RR and pre-RROA spectra (λ_{ex}=785 nm) of AmB in water at pH=12.7 S7
Figure S9. Calculated conformational changes of AmB in DMSO S8
Figure S10. Simulated Raman and ROA spectra of AmB in DMSO S8
Figure S11. Effects of different excitation wavelengths on the simulated Raman and ROA spectra of AmB in DMSO S9
Table S2. Dissymmetry factor values obtained from AmB experimental spectra S9
Table S3. Circular intensity difference values obtained from the AmB experimental spectra S10
References S10

Table S1. Set of Raman and ROA measurement parameters of AmB solutions.

<table>
<tr><th rowspan="2">solvent</th><th rowspan="2">concentration [mol/dm^3]</th><th colspan="2">λ_{ex}= 532 nm</th><th colspan="2">λ_{ex}= 785 nm</th></tr>
<tr><th>laser power [mW]</th><th>total data collection time [h]</th><th>laser power [mW]</th><th>total data collection time [h]</th></tr>
<tr><td>DMSO</td><td>8x10^{-4}</td><td>20</td><td>66</td><td rowspan="4">200</td><td>28</td></tr>
<tr><td>a mixture of DMSO/MeOH in 1:1 (v/v) ratio</td><td>8x10^{-4}</td><td>20</td><td>58</td><td>53</td></tr>
<tr><td>water at pH=12.7</td><td>4x10^{-3}</td><td>30</td><td>56</td><td>15</td></tr>
<tr><td>water at pH=12.7</td><td>8x10^{-3}</td><td>20</td><td>57</td><td>50</td></tr>
</table>

ECD-Raman correction

The ECD-Raman was calculated and subtracted from measured ROA spectra according to the previously published procedure[1,2] using the formula:

$$I^{SCP}_{ECD-RAM}(L',L) =$$
$$\frac{1}{2}\left[I^{R}_{dif}(L',L)\Delta\varepsilon + I^{R}_{sum}(L',L)\Delta\varepsilon'\right]\frac{[1+(\varepsilon+\varepsilon')C\ln 10\, L'](1-10^{-(\varepsilon+\varepsilon')CL}) - (\varepsilon+\varepsilon')C\ln 10 L\, 10^{-(\varepsilon+\varepsilon')CL}}{(\varepsilon+\varepsilon')(1-10^{-(\varepsilon+\varepsilon')CL})}$$

where the $\Delta\varepsilon/\varepsilon$ and $\Delta\varepsilon'/\varepsilon'$ are ECD/UV-vis intensities at the incident and scattered light frequency; $I^R_{dif} = I^R_R - I^R_L$ and $I^R_{sum} = I^R_R + I^R_L \cong I^{SCP}_{RAM}$ (I^i_s is a scattered light intensity, characterised by the incident *i* and the scattered *s* polarization states); *L* (cm) is the pathlength of the focal range of the spectrometer where both ROA/Raman and ECD/UV-vis absorption are active; *L'* (cm) is the ECD/UV-vis absorption active pathlength; *C* (mol/L) is a molar concentration of an absorbing solute.

Here, for calculations of the ECD-Raman of AmB, we used *L*=1.5 and *L'*=0 cm.

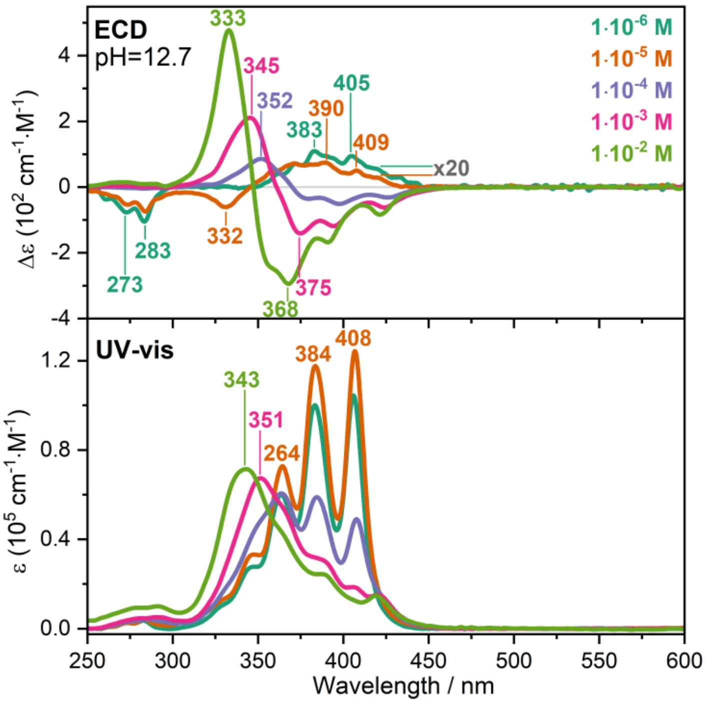

Figure S1. Comparison of ECD (upper panel) and UV-vis (lower panel) spectra of AmB species dissolved in water at pH=12.7. Several concentrations of AmB were used (from 1x10^{-6} M to 1x10^{-2} M). All electronic spectra were recorded immediately after the sample preparation. ECD signals of AmB measured for the concentration of 1x10^{-6} M and 1x10^{-5} M were multiplied by 20 for clarity.

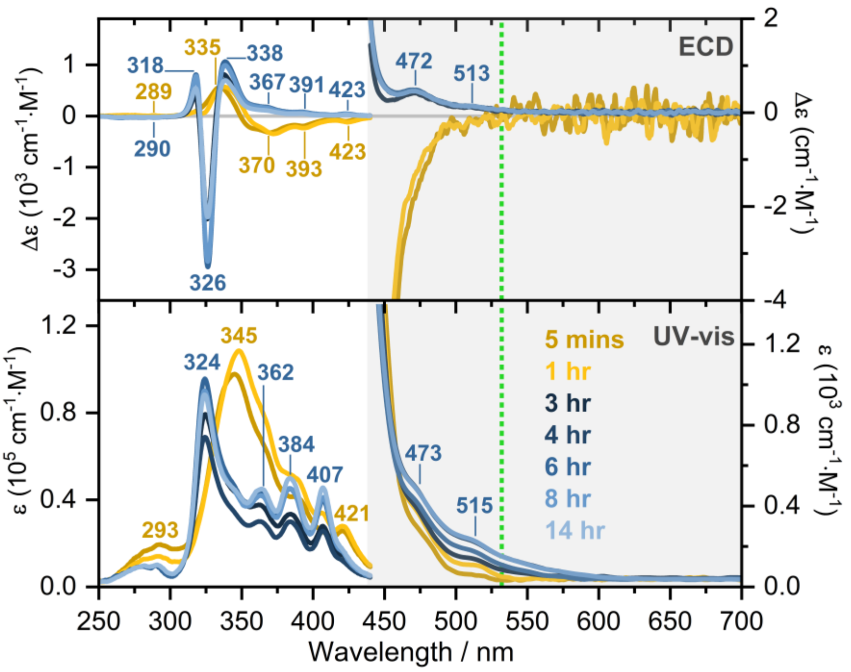

Figure S2. Comparison of ECD (upper panel) and UV-vis (lower panel) spectra obtained for AmB species in water at pH=12.7 and a concentration of 4x10^{-3} M. The electronic spectra were measured after 5 minutes, 1, 3, 4, 6, 8, and 14 hours of the sample preparation. The green dotted line indicates the excitation wavelength used to measure RR and RROA spectra (532 nm).

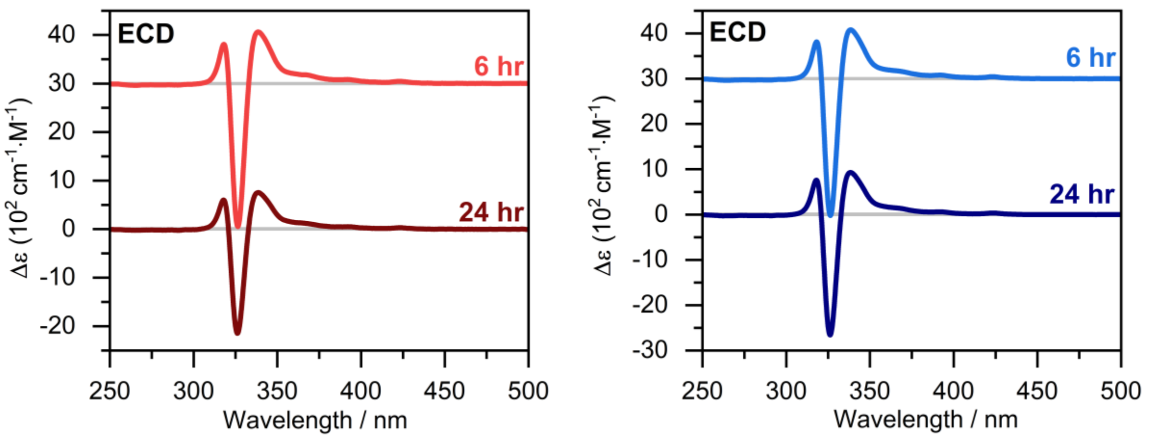

Figure S3. Comparison of ECD spectra for AmB species forming in water medium at pH=12.7 at two different concentrations: 4x10^{-3} M (left panel) and 8x10^{-3} M (right panel). The ECD spectra were recorded after 6 and 24 hours of sample preparation.

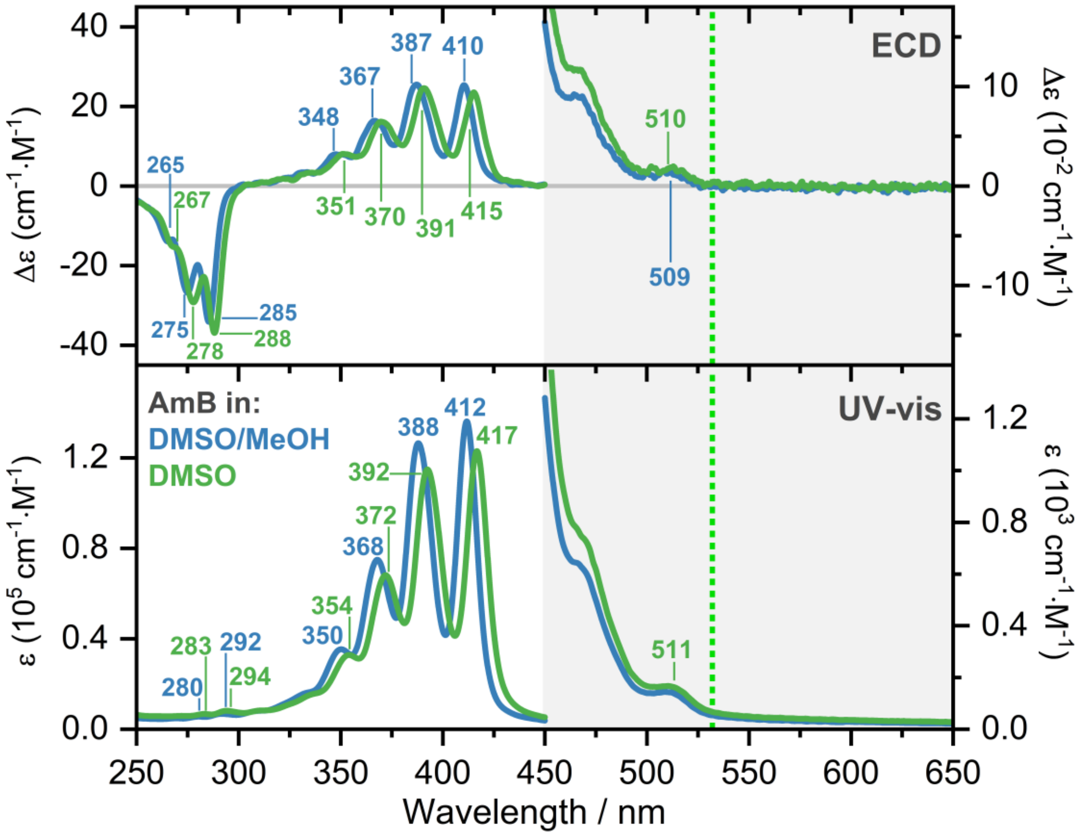

Figure S4. Comparison of ECD (upper panel) and UV-vis (lower panel) spectra of AmB in DMSO (c=8x10^{-4} M) and a mixture of DMSO/MeOH in a 1:1 (v/v) ratio (c=8x10^{-4} M). The electronic spectra were recorded immediately after the sample preparation.

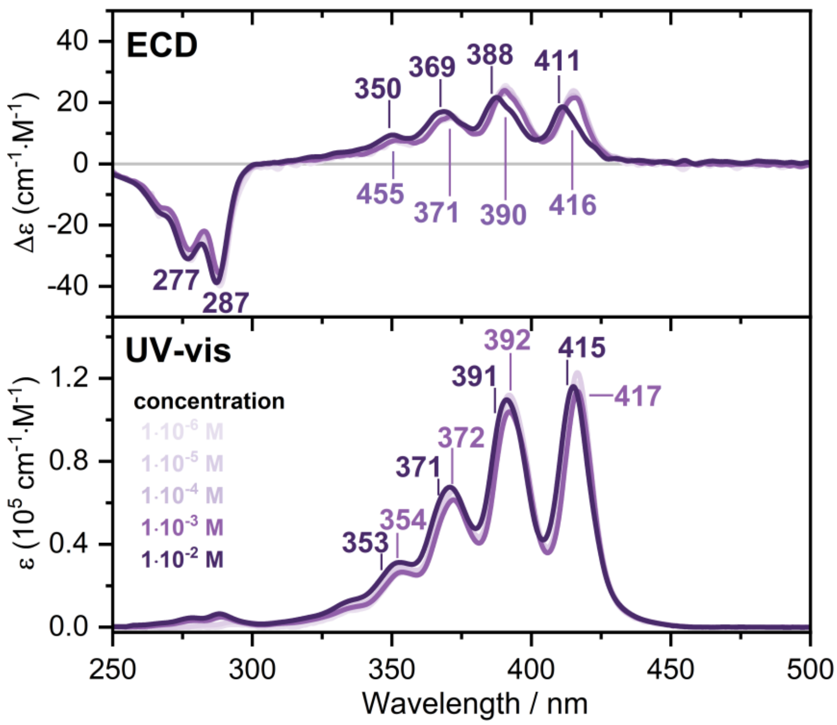

Figure S5. Comparison of ECD (upper panel) and UV-vis (lower panel) spectra of AmB species in DMSO. Several concentrations of AmB were used (from $1x10^{-6}$ to $1x10^{-2}$ M). The electronic spectra were measured immediately after the sample preparation.

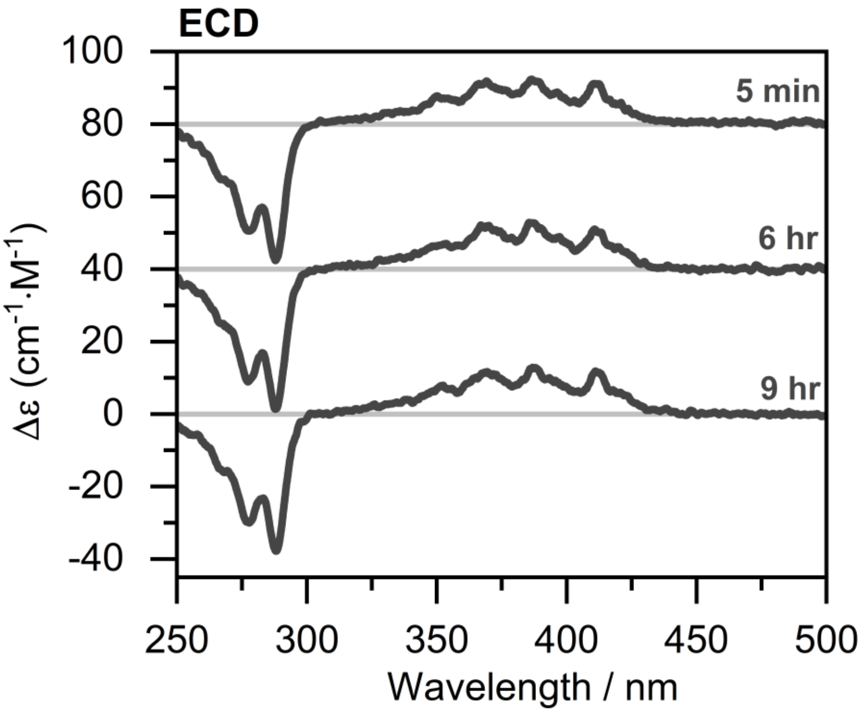

Figure S6. Comparison of ECD spectra for AmB in DMSO at a concentration of $8x10^{-4}$ M. The ECD spectra were recorded after 5 minutes, 6 and 9 hours of the sample preparation.

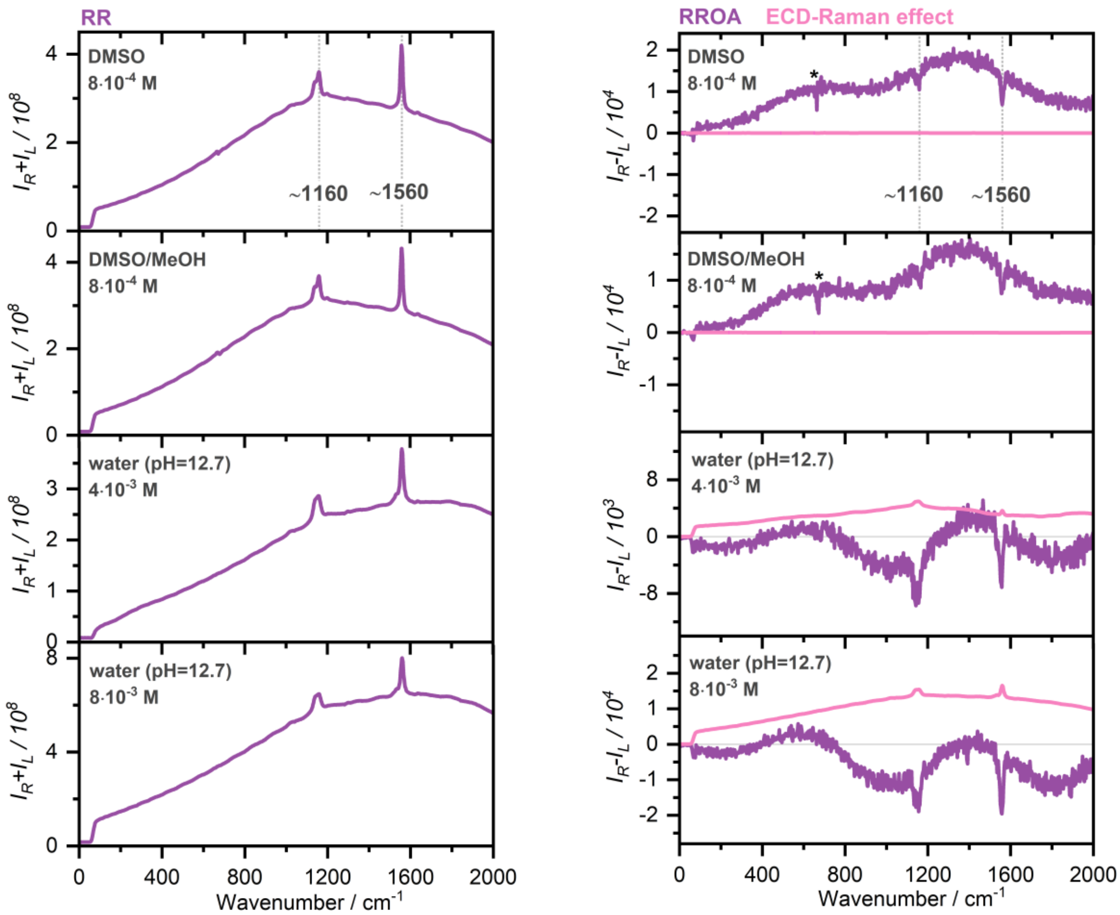

Figure S7. Comparison of solvent-corrected RR (left panel) and RROA (right panel) spectra of AmB dissolved in organic and inorganic solvents. All spectra were obtained with an excitation wavelength of 532 nm. The RROA spectra are after ECD-Raman correction. Asterisks mark the solvent's artifacts on RROA spectra. The RR/RROA spectra of AmB in alkaline water were recorded after 6 hours of sample preparation.

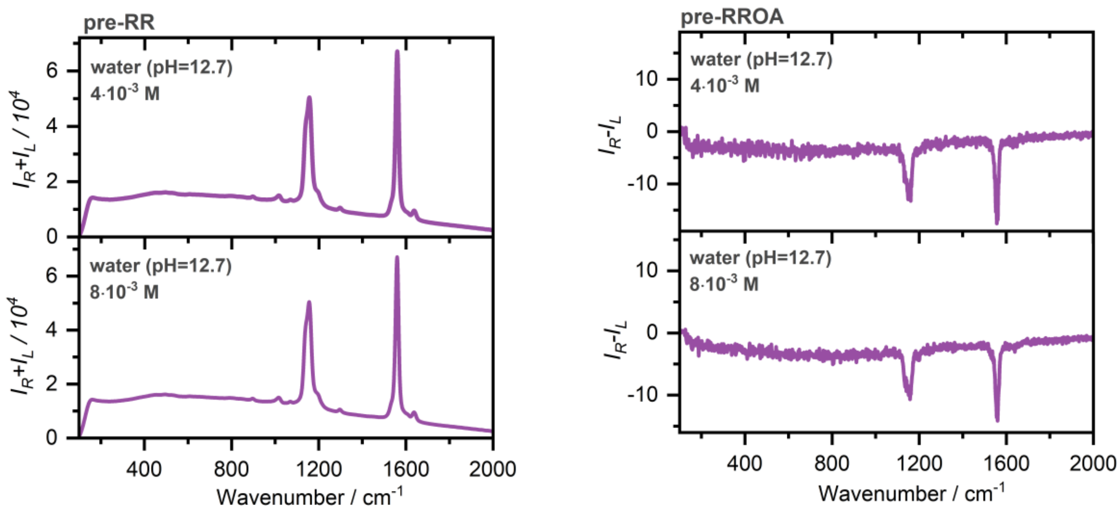

Figure S8. Comparison of solvent-corrected pre-RR (left panel) and pre-RROA (right panel) spectra of AmB dissolved in an alkaline medium. All spectra were obtained with an excitation wavelength of 785 nm after 6 hours of sample preparation.

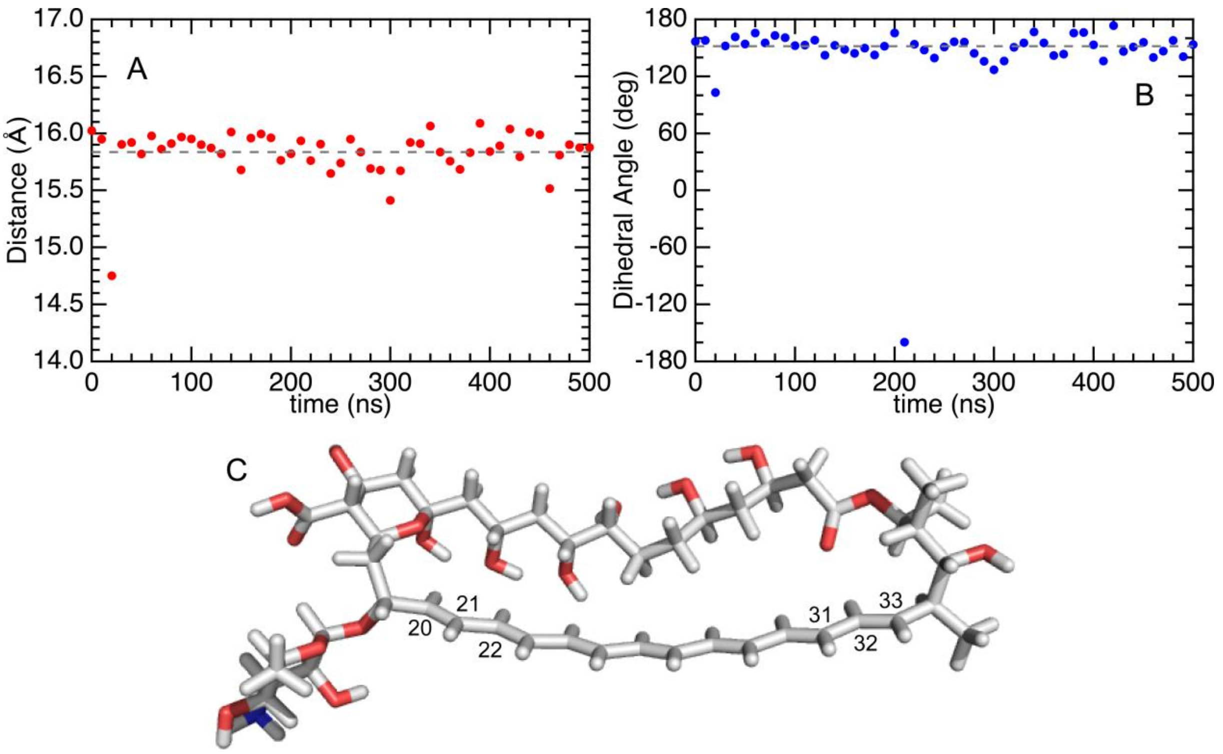

Figure S9. Conformational changes of AmB in DMSO. (A) The distances between the C20 and C33 atoms, as well as the dihedral angle between the two planes defined by the C20-C21-C22 and C31-C32-C33 atoms, are plotted for the 51 QM/MM optimized structures. The gray dashed lines indicate the corresponding average values of 15.8 Å and 151.7° for panels A and B, respectively. (C) Representative optimized geometry of AmB. The structure is based on an MD snapshot at 270 ns. Gray, blue, and red represent carbon, nitrogen, and oxygen atoms, respectively. Hydrogen atoms are also illustrated in gray. Important carbon atom numbers are shown.

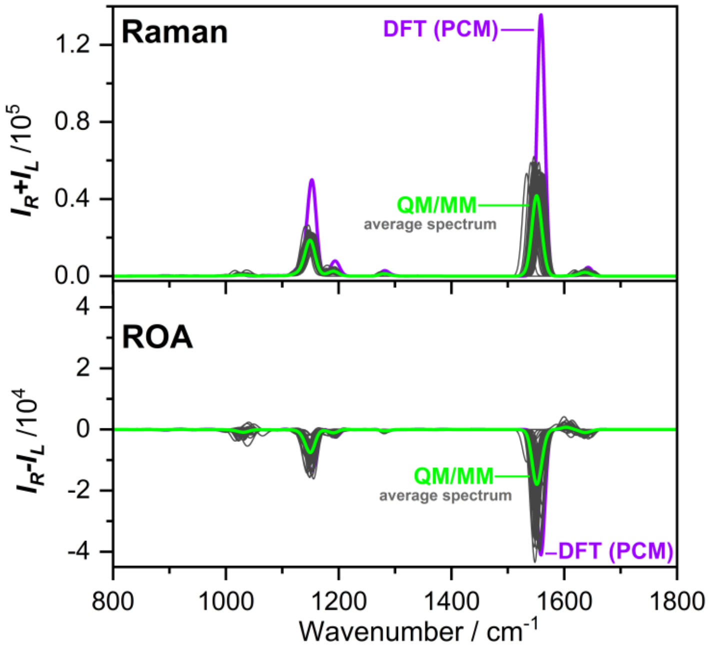

Figure S10. Simulated Raman and ROA spectra of AmB in DMSO. The spectra are averaged over 51 MD solute-solvent clusters and were obtained with an excitation line of 785 nm.

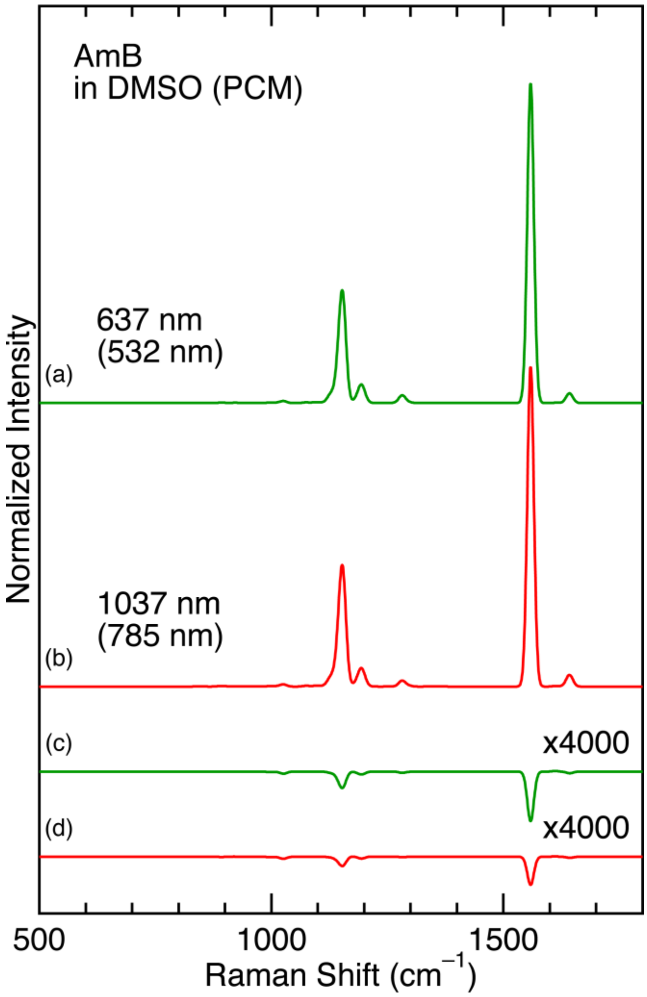

Figure S11. Effects of different excitation wavelengths on the simulated Raman and ROA spectra of AmB in DMSO using the CPCM polarizable conductor model. The excitation wavelengths of 637 and 1037 nm correspond to the experimental 532 and 785 nm, respectively, because of a red shift of the theoretical electronic transition of interest to the experimental spectra. (c, d) The ROA intensities are magnified by a factor of 4000.

Table S2. Dissymmetry factor (*g*-factor, ECD/UV-vis) values, plotted for selected bands, obtained from experimental spectra of AmB species in different solvents.

DMSO **c=8x10^{-4} M**		DMSO/MeOH **c=8x10^{-4} M**		water pH=12.7 **c=4x10^{-3} M**		water pH=12.7 **c=8x10^{-3} M**	
**λ / nm**	***g***	**λ / nm**	***g***	**λ / nm**	***g***	**λ / nm**	***g***
372	2.5x10^{-4}	368	2.2x10^{-4}	326	-3.2x10^{-2}	326	-3.3x10^{-2}
392	2.1x10^{-4}	388	2.0x10^{-4}	338	2.0x10^{-2}	338	2.1x10^{-2}
417	2.0x10^{-4}	412	1.9x10^{-4}	391	2.3x10^{-3}	392	3.1x10^{-3}
511	1.4x10^{-4}	511	1.4x10^{-4}	423	3.2x10^{-3}	423	3.5x10^{-3}
---	---	---	---	472	9.0x10^{-3}	471	1.7x10^{-3}
---	---	---	---	513	6.6x10^{-3}	513	1.5x10^{-3}

Table S3. Circular intensity difference (CID, the ratio of ROA to Raman intensity) values, plotted for selected vibrational bands, obtained from AmB experimental spectra.

DMSO **$8x10^{-4}$ M**				DMSO/MeOH **$8x10^{-4}$ M**			
**785 nm**		**532 nm**		**785 nm**		**532 nm**	
**v / cm^{-1}**	**CID**	**v / cm^{-1}**	**CID**	**v / cm^{-1}**	**CID**	**v / cm^{-1}**	**CID**
1561	-1.4x10^{-4}	1560	-4.7x10^{-5}	1560	-1.1x10^{-4}	1558	-3.0x10^{-5}
1155	-1.4x10^{-4}	1157	-5.7x10^{-5}	1160	-1.3x10^{-4}	1160	-4.3x10^{-5}
1134	-5.5x10^{-5}	1141	-5.9x10^{-5}	1149	-7.4x10^{-5}	1143	-3.5x10^{-5}
Water pH=12.7 **$4x10^{-3}$ M**				Water pH=12.7 **$8x10^{-3}$ M**			
**785 nm**		**532 nm**		**785 nm**		**532 nm**	
**v / cm^{-1}**	**CID**	**v / cm^{-1}**	**CID**	**v / cm^{-1}**	**CID**	**v / cm^{-1}**	**CID**
1559	-2.5x10^{-4}	1558	-7.1x10^{-5}	1557	-1.7x10^{-4}	1558	-9.8x10^{-5}
1156	-2.6x10^{-4}	1155	-1.3x10^{-4}	1155	-1.5x10^{-4}	1155	-1.5x10^{-4}
1143	-1.3x10^{-4}	1143	-1.4x10^{-4}	1137	-1.9x10^{-4}	1143	-1.3x10^{-4}

References

(1) Wu, T.; Kapitán, J.; Bouř, P. Resolving Resonant Electronic States in Chiral Metal Complexes by Raman Optical Activity Spectroscopy. *J. Phys. Chem. Lett.* **2022**, *13* (17), 3873–3877.

(2) Machalska, E.; Zajac, G.; Wierzba, A. J.; Kapitán, J.; Andruniów, T.; Spiegel, M.; Gryko, D.; Bouř, P.; Baranska, M. Recognition of the True and False Resonance Raman Optical Activity. *Angew. Chem. Int. Ed.* **2021**, *60* (39), 21205–21210.
